# *Granulicatella adiacens* and *Abiotrophia defectiva* Native Vertebral Osteomyelitis: Three Cases and Literature Review of Clinical Characteristics and Treatment Approach

**DOI:** 10.1155/2019/5038563

**Published:** 2019-05-06

**Authors:** Cinzia Puzzolante, Gianluca Cuomo, Marianna Meschiari, Andrea Bedini, Aurora Bonazza, Claudia Venturelli, Mario Sarti, Cristina Mussini

**Affiliations:** ^1^Azienda Ospedaliero-Universitaria di Modena, Infectious Disease Clinic, Modena, Italy; ^2^Clinical Microbiology, Azienda Ospedaliero-Universitaria di Modena, Modena, Italy; ^3^Clinical Microbiology, Ospedale Civile di Baggiovara, Modena, Italy; ^4^University of Modena and Reggio Emilia, Azienda Ospedaliero-Universitaria di Modena, Infectious Disease Clinic, Modena, Italy

## Abstract

*Granulicatella adiacens* and *Abiotrophia defectiva* are an increasingly recognized cause of osteoarticular infections. We describe two cases of *G. adiacens* and one case of *A. defectiva* native vertebral osteomyelitis (NVO) and review all published cases. Nine cases of *G. adiacens* NVO and two cases of *A. defectiva* NVO were previously described. Patients were usually middle-aged men, and classical risk factors for NVO were present in half of the cases. Concomitant bacteremia was reported in 78.6% of cases, and concurrent infective endocarditis occurred in 36.4% of this sub-group of patients. Many different antibiotic schemes were recorded, with median treatment duration of 6 weeks. In the most recent reports, glycopeptides represented the most frequent empirical therapy, possibly due to the increasing emergence of *G. adiacens* and *A. defectiva* penicillin-resistant strains. Stabilization surgery was rarely required (14.3% of cases), and clinical cure was generally achieved. In conclusion, *Granulicatella spp*. and *Abiotrophia spp*. NVO is rare but increasingly described. A total antibiotic course of six weeks seems to be appropriate for noncomplicated cases, and clinical outcome is generally favorable.

## 1. Introduction and Inclusion Criteria for Case Definition

*Granulicatella* species and the related genus *Abiotrophia* are Gram-positive lactic acid bacteria, previously referred to as nutritionally variant streptococci because of their requirement of L-cysteine or pyridoxal into culture media for growth [1]. *Granulicatella* (formerly *Abiotrophia*) *adiacens*, *Abiotrophia defectiva*, and *Granulicatella elegans* are parts of the normal oropharyngeal, gastrointestinal, and urogenital microflora but might also act as opportunistic pathogens [2]. Recently, thanks to 16S rRNA sequence analysis, *Granulicatella para-adiacens* (a species closely related to *G. adiacens*) has also emerged as pathogenic for humans [3].

Usually considered a rare cause of infective endocarditis (IE), *G. adiacens, G. elegans,* and *A. defectiva* account for 5–16% of all streptococcal IE cases [4, 5], even if the challenge in identification of these fastidious organisms may lead to underestimate the real incidence. Indeed, the pathogenic role of *G. adiacens* and *A. defectiva* in osteoarticular infections such as native vertebral osteomyelitis or spondylodiscitis [6–15], prosthetic-related infections [16, 17], and septic arthritis [18] is increasingly recognized.

Here, we described two cases of *G. adiacens* and one case of *A. defectiva* native vertebral osteomyelitis occurring in our institution between 1 January 2008 and 1 December 2018. We also reviewed all cases of native vertebral osteomyelitis due to these organisms described in the medical literature. We searched PubMed articles written in English between 1 January 1990 and 1 December 2018 using a combination of the following key words: “*granulicatella*,” “*abiotrophia*,” “nutritionally variant streptococci,” “spondylodiscitis,” and “vertebral osteomyelitis.” Moreover, in the Discussion section, we summarize applicable guidelines and recent data about *G. adiacens* and *A. defectiva* antibiotics susceptibility, focusing on molecules of clinical utility in the setting of bone infections.

## 2. Presentation of Institutional Cases

### 2.1. Case 1

In April 2017, a 50-year-old man with irrelevant past medical history started to report nocturnal low-grade fever and low back pain. He empirically received a short course of antibiotics but fever occasionally relapsed. During the following weeks, the patient experienced progressive dyspnea that led him to the local emergency department (ED). At the arrival in the ED, the patient was febrile (38.2°C), and his laboratory exams showed marked leukocytosis (WBC = 22.9 G/*μ*L, 77% neutrophils), mild anemia (Hb = 10.6 g/dL), and increased C-reactive protein (CRP = 11.2 mg/dL).

**Figure 1:**
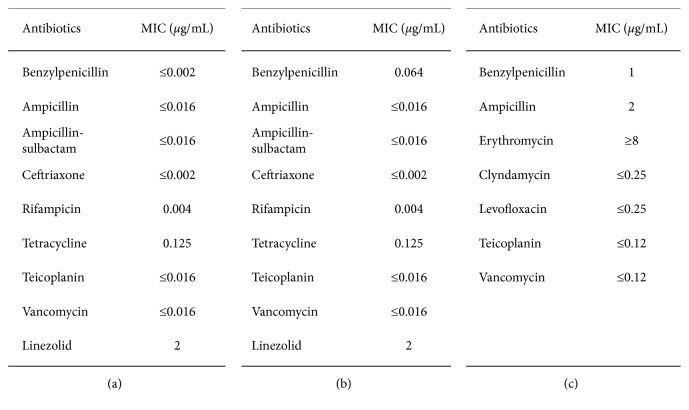
Antibiograms and MICs of *Granulicatella adiacens* and *Abiotrophia defectiva* strains isolated from the clinical cases. (a) Case 1 *G. adiacens* strain MICs. (b) Case 2 *G. adiacen*s strain MICs. (c) Case 3 *A. defectiva* strain MICs.

A transthoracic echocardiography showed a massive aortic insufficiency with evidence of multiple vegetations on the free edge of the aortic cuspids. Two sets of blood cultures were performed in the ED, and *G. adiacens* grew both from aerobic and anaerobic blood bottles after 17 and 21 hours in the first set and after 17 and 22 hours in the second set, respectively. Blood cultures (BACT/ALERT FA Plus and BACT/ALERT FN Plus) were processed using the BACT/ALERT system (bioMèrieux). Agar MHF for fastidious organism plates was incubated in 5% CO_2_ at 35–37°C for 48 hours. Identification was carried out by matrix-assisted laser desorption ionization-time of flight mass spectrometry (MALDI-TOF MS) using the Vitek MS system (bioMerieux). Antimicrobial susceptibility was performed by E-test method, and MICs were reported according to PK-PD (nonspecies related) breakpoints as stated in March 2017 EUCAST Clinical Breakpoints Tables [19] (Figure 1(a)). Three days after ED admission, reparative aortic surgery was performed in a local cardiac surgery unit. Subsequently, the patient underwent a CT scan of the spine for persistent back pain, and a diagnosis of L3-L4 native vertebral osteomyelitis was made. The patient was transferred in May 2017 to our clinic to continue the antibiotic course. MRI of the spine confirmed the L3-L4 native vertebral osteomyelitis and showed similar degenerative changes also in L5-S1. The patient received a total of two-week intravenous course of vancomycin 2 g/daily + ceftriaxone 2 g/daily + gentamicin 5 mg/kg/daily, followed by intravenous ampicillin 12 g/daily for two weeks. Patient's clinical conditions improved, and he was discharged with oral amoxicillin 4 g/daily for two weeks. A six-month follow-up MRI of the lumbar spine showed an initial healing of the infectious process, and the patient reported a significantly improvement of low back pain as well.

### 2.2. Case 2

A 47-year-old man was admitted to our clinic on May 2017 with a 10-day history of fever and severe low back pain after returning from a scuba diving session in Maldives. His past medical history included hypertension and chronic back pain due to L5 disc herniation. On examination, body temperature was 38.5°C and blood pressure was 110/80 mmHg. Laboratory results showed a normal white cell formula (WBC = 10.3 G/*μ*L, 79% neutrophils) and a raise of C-reactive protein (CRP = 16.5 mg/dL). Two sets of blood cultures were performed at admission, and *G. adiacens* grew both from aerobic and anaerobic blood bottles after 15 and 18 hours in the first set and after 16 hours in both aerobic and anaerobic bottles of the second set. Blood cultures (Bactec Plus Aerobic/F and Bactec Plus Anaerobic/F) were processed using the BACTEC FX system (Becton Dickinson). Identification and antimicrobial susceptibility (Figure 1(b)) were carried out as in Case 1.

**Figure 2:**
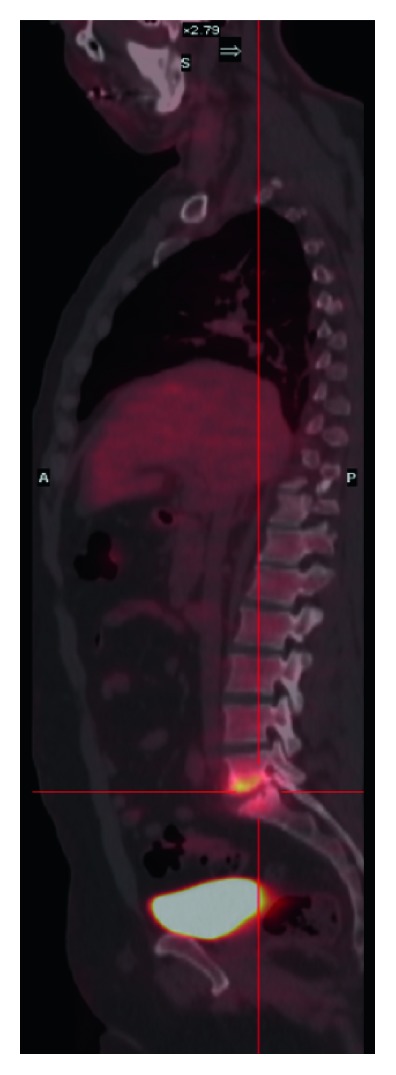
FDG-PET/CT scan of patient 2 showing an intense metabolic uptake between the lower plate of L5 and the upper plate of S1.

A transthoracic echocardiography was negative for IE. MRI of the spine showed an increased STIR signal change at the L5-S1 level suggestive for early spondylodiscitis. To determine if the morphostructural bone changes described in MRI were metabolically active, a total body FDG-PET/CT scan was performed: an intense L5-S1 standardized uptake value (SUV = 7.1) was detected (see Figure 2) and diagnosis of native vertebral osteomyelitis was made; no other metabolically active areas were detected. The patient started empirically iv vancomycin 2 g/daily plus ceftriaxone 2 g/daily for one week, and then switched to ceftriaxone alone for 3 weeks. At discharge, the patient was switched to oral amoxicillin 3 g/daily for 2 weeks. A three-month clinical follow-up was uneventful, with gradual pain reduction. The patient fully recovered; no follow-up MRI was performed.

### 2.3. Case 3

In October 2018, a 75-year-old woman with previous mitral valvuloplasty and previous breast cancer was admitted in our clinic for persistent low back pain started three months earlier without fever. An MRI of the spine performed two days before admission showed a L4-L5 infectious process. At the admission, blood tests showed a mild anemia (Hb = 9.5 g/dL) and a mild elevation of CRP (CRP = 2.5 mg/dL). A chest CT scan revealed a right pleural effusion with bilateral parenchymal consolidation; a diagnostic thoracentesis was negative for microbial growth. At day 15, the patient became feverish, and two sets of blood cultures were performed. *A. defectiva* grew both from aerobic and anaerobic blood bottles after 27 and 28 hours in the first set and after 70 and 34 hours in the second set, respectively. Blood cultures processing, microbiological identification, and antimicrobial susceptibility were carried out as in Case 1. The isolated *A. defectiva* strain showed a reduced penicillin and ampicillin susceptibility (Figure 1(c)); thus, a glycopeptide-based antibiotic regimen was started. A transthoracic echocardiography showed a severe mitral insufficiency without evidence of vegetations. The patient refused to perform a transesophageal echocardiography. A FDG-PET/CT scan confirmed a localized metabolic uptake at the L4-L5 level (SUV = 3.5); no other metabolically active areas were detected.

The patient received initially iv vancomycin 2 g/daily, and then, she was transferred to a local cardiac surgery unit for mitral valve replacement. Because of initial renal failure, after two weeks, the patient was switched to teicoplanin 400 mg/daily according to the local infectious diseases specialist's consultation. Mitral valve culture was negative for microbial growth. Surgical follow-up was uneventful, and after three weeks, the patient was transferred to a cardiac rehabilitation unit, where she completed a six-week course of iv teicoplanin. At a six-month follow-up visit, the patient reported an initial improvement of low back pain; no follow-up MRI was performed.

## 3. Clinical Characteristics of *Granulicatella adiacens* and *Abiotrophia defectiva* Native Vertebral Osteomyelitis

During a ten-year period, we recorded in our institution two cases of native vertebral osteomyelitis due to *G. adiacens* and one due to *A. defectiva.* Other eleven cases of native vertebral osteomyelitis due to these fastidious organisms were identified from PubMed search: nine due to *G. adiacens* [6–13] and two due to *A. defectiva* [14, 15]. At the present, no cases of *G. elegans* or *G. para-adiacens* spondylodiscitis have been described.

*G. adiacens* and *A. defectiva* vertebral osteomyelitis clinical characteristics and therapeutic regimens, including our three cases, are shown in Table 1. Patients were predominantly males (78.5%) with a median age of 50.5 years (IQR = 47.7–63.5). Many patients (8/14, 57.1%) presented identifiable risk factors for native vertebral osteomyelitis such as injecting drug use, infective endocarditis, degenerative spinal disease, and diabetes mellitus.

As expected, all patients reported back pain; fever was recorded in 9/14 cases (64.3%). Most nutritionally, variant streptococci native vertebral osteomyelitis cases were associated with bacteremia (11/14 cases, 78.6%); meanwhile, concurrent IE occurred only in a subgroup of these patients (4/11, 36.4%).

Twelve different therapeutic schemes were recorded; median antibiotic therapy duration was six weeks (IQR = 6-7), ranging from to 4 to 15 weeks. Surgical stabilization was required in 2 out of 14 cases (14.3%). When outcome was described (12 patients), clinical cure was achieved in all cases.

## 4. Discussion and Conclusions

Once known as nutritionally variant streptococci, *G. adiacens* and *A. defectiva* are a rare cause of native vertebral osteomyelitis. Discrepancies still exist in terms of correct taxonomic classification, being *Granulicatella* and *Abiotrophia* terms used interchangeably even in recent articles [9, 10, 13].

As expected, the clinical presentation of *G. adiacens* and *A. defectiva* native vertebral osteomyelitis was back pain, usually associated with fever. Patients with *Granulicatella* and *Abiotrophia* spondylodiscitis were usually in their fifth decade or older, and approximately half of cases presented classical risk factors for spondylodiscitis such as endocarditis, intravenous drug using, or immunosuppressive conditions. On the other hand, in a recent analysis, including 38 cases of *G. adiacens* endocarditis and 38 cases of *A. defectiva* endocarditis, systemic embolism excluding the central nervous system occurred in 9.4% and 11.8% of patients for each group, respectively [5].

Usually, microbiological diagnosis of *G. adiacens* and *A. defectiva* native vertebral osteomyelitis is made through blood culturing and/or bone biopsy. Automatic biochemical test systems, such as Vitek 2, are widely used in clinical practice, but phenotypic characteristics alone may be inaccurate in *Granulicatella spp.* and *Abiotrophia spp.* identification [20]. After the advent of 16S rRNA gene sequencing and MALDI-TOF MS, identification within this group of fastidious bacteria have been made more readily and accurately performed both at genus and species levels [21].

At the present, European EUCAST guidelines do not provide specific recommendations on susceptibility testing and MICs interpretation for *Granulicatella* and *Abiotrophia* species and suggest the usage of PK-PD (nonspecies related) breakpoints avoiding interpretation on susceptibility [19]. As the vast majority of the European microbiology laboratories, our clinical microbiology laboratory follows EUCAST guidelines, and disk diffusion tests and E-tests are routinely performed for MIC determinations; when indicated and in case of multidrug-resistant organisms, broth microdilution method is used. On the contrary, US CLSI guidelines, through the M45 Document on infrequently isolated or fastidious bacteria [22], give indications on susceptibility testing and suggest interpretative criteria for this class of microorganisms. Indeed, most of the available data for *Granulicatella spp*. and *Abiotrophia spp*. are based on CLSI indications. In particular, using broth microdilution for MIC determination as suggested in CLSI guidelines, differences in terms of antimicrobial susceptibilities between the two genera recently emerged [23–26]. In these studies, susceptibility ranged from 34 to 39% for penicillin, from 22 to 47% for ceftriaxone, and from 3 to 83% for cefepime for *G. adiacens* strains. *A. defectiva* strains resulted less susceptible to penicillin (range 10.8–24%) but more susceptible to ceftriaxone (range 92–100%). On the contrary, the vast majority of *Granulicatella* spp. and *A. defectiva* isolates were fully susceptible to vancomycin, clindamycin, meropenem, and levofloxacin.

Thus, in the setting of *A. defectiva* native vertebral osteomyelitis, a third-generation cephalosporin is still an appropriate empiric therapy; meanwhile in case of *G. adiacens* native vertebral osteomyelitis, an alternative parenteral agent such as a glycopeptide should be used while antimicrobial susceptibility testing is pending. Agents with high oral availability such as levofloxacin and clindamycin may represent a good option for oral switch. Few data on rifampin, daptomycin, and linezolid are available, and there are no CLSI or EUCAST interpretive breakpoints for these molecules. Elevated MIC50 and MIC90 for daptomycin and linezolid were found in two different studies including overall more than four hundred *G. adiacens* and more than one hundred fifty *A. defectiva* isolates [23, 26]; therefore, these two molecules should not currently be considered an option.

Given the limited microbiological and clinical data and a potential higher risk of complications, the American Heart Association (AHA) and the European Society of Cardiology (ESC) guidelines recommend a 4- to 6-week course of antibiotics for the treatment of endocarditis due to *G. adiacens* and *A. defectiva* [27, 28]. The suggested regimen combines penicillin *G*, ampicillin, or ceftriaxone plus an aminoglycoside for at least the first two weeks or vancomycin alone in case of beta-lactam allergy as for enterococci infection. The “2015 Native Vertebral Osteomyelitis IDSA Guidelines” do not expressly focus on nutritionally variant streptococci, but antibiotic treatment can be similarly inferred from enterococci and streptococcal infections, which in turn do not substantially differ from AHA and ESC recommendations [29]. Of note, in the first case reports, initial antibiotic therapy was penicillin based; in the last years, we observed a greater tendency to prescribe a glycopetide-based antibiotic regimen as empirical or definitive therapy. This may reflect the increasing reporting of penicillin-resistant *G. adiacens* strains. In our two cases, *G. adiacens* strains were susceptible to penicillins; thus, after an initial empirical parenteral therapy with vancomycin and ceftriaxone (plus gentamicin in the case with IE), oral therapy with amoxicillin was prescribed at discharge to conclude the antimicrobial course.

Radiological work-up of NVS native vertebral osteomyelitis generally included MRI imaging. In our patient 1, MRI demonstrated a second infectious spine focus not detected from a previous CT scan. However, in early stages of native vertebral steomyelitis, bone and surrounding tissue changes seen in MRI may be aspecific: in these cases, FDG-PET/CT scan may be helpful to discriminate between a chronic degenerative bone alteration and an early infectious process [30], as well as in our patient two. In this latter case, a negative transthoracic echocardiography along with the absence of FDG-PET/CT uptakes in other sites, gave us a low clinical suspicion of IE, and a transesophageal echocardiography was not performed. Considering cases reported in the literature, treatment duration ranged from 4 to 15 weeks. In our cases as well as in the most recent articles, *G. adiacens* native vertebral osteomyelitis treatment duration has been set to six weeks, presumably influenced both by the previous cited 2015 native vertebral osteomyelitis IDSA guidelines, and Bernard et al. randomized clinical trial on pyogenic native vertebral osteomyelitis treatment duration [31]. This seems not to have affected the general favorable clinical outcome observed in all cases, especially because complications as paravertebral abscesses or vertebral instability requiring surgery occurred in two cases only.

In conclusions, *Granulicatella* and *Abiotrophia* species are a rare but increasingly more recognized cause of osteoarticular infections, including native vertebral osteomyelitis. Blood isolation of the causative organism is frequent in this setting, and native vertebral osteomyelitis may occur in the absence of infective endocarditis. Considering data about *Granulicatella* spp. and *Abiotrophia* spp. reduced susceptibility to penicillins and cephalosporins, a glycopeptide-based regimen may represent a therapeutic option while antimicrobial susceptibility testing is pending. Clinical outcome is generally favorable, and noncomplicated cases of *Granulicatella adiacens* and *Abiotrophia defectiva* spondylodiscitis can be effectively treated with a standard six-week course of antibiotic therapy.

## Figures and Tables

**Table 1:** Clinical characteristics, antibiotic treatment, and outcome of *Granulicatella adiacens* and *Abiotrophia defectiva* NVO cases.

Reference (year of publication)	Patient age, sex	Clinical presentation	Past medical history	Concomitant endocarditis	Bacteremia	Vertebral infection site	Positive microbiological specimens	Methods for microbial identification	Microbiological identification	Antibiotic regimen	Total duration of treatment	Spinal surgical intervention	Outcome
Our first case (2017)	50, male	Cardiogenic shock, fever, back pain	None	Yes	Yes	L3-L4, L5-S1	Blood cultures	Vitek MS	*Granulicatella adiacens*	Vancomycin + ceftriaxone + gentamycin for 2 weeks, ceftriaxone for 2 weeks, amoxicillin po for 2 weeks	6 weeks	No	Cured
Our second case (2017)	47, male	Fever, back pain	Hypertension, L5 disc herniation	No	Yes	L5-S1	Blood cultures	Vitek MS	*Granulicatella adiacens*	Vancomycin + ceftriaxone for 1 week, ceftriaxone for 3 weeks, amoxicillin po for 2 weeks	6 weeks	No	Cured
Our third case (2018)	75, female	Back pain	Previous mitral valvuloplasty	No	Yes	L4-L5	Blood cultures + bone biopsy	Vitek MS	*Abiotrophia defectiva*	Vancomycin for 2 weeks, teicoplanin for 6 weeks	8 weeks	No	NA
[15] (2017)	54, female	Fever, back pain	Patent ductus arteriosus	No (pulmonary artery endoarteritis)	Yes	L5-S1	Blood cultures	Vitek 2 system + 16S rNA sequencing	*Abiotrophia defectiva*	Penicillin *G* + gentamicin	Planned 6 weeks	No	NA
[13] (2017)	48, male	Back pain	Intravenous drug abuse, mitral valve prolapse, HCV infection	No	No	L3-L4 + L3-L5 epidural phlegmon	Vertebral, disk and paraspinal muscles biopsy	ND	*Granulicatella/Abiotrophia spp.*	Vancomycin for 6 weeks + cefepime in the first day	6 weeks	No	Cured
[12] (2017)	61, male	Back pain	Diabetes, hypertension	No	No	L3-L4	Disk biopsy	Vitek 2 system	*Granulicatella adiacens*	Ceftriaxone + gentamycin for 6 weeks	6 weeks	No	Cured
[11] (2016)	46, male	Back pain	Diabetes, recent dental procedure	No	No	L2 inferior endplate	Bone biopsy	PCR (not specified)	*Granulicatella adiacens*	Vancomycin for 6 weeks	6 weeks	Yes (L1-L3 fixation and interbody cage position in L2)	Cured
[10] (2015)	62, male	Back pain, fever	Hypertension	No	Yes	T10-T12 with spinal abscess	Blood cultures	ND	*Abiotrophia adiacens*	Vancomycin for 6 weeks	6 weeks	Yes (laminectomy with T9-L2 fusion surgery)	Cured
[9] (2013)	48, female	Back pain, fever	Parkinson's disease	No	Yes	L3-L5	Blood cultures + disk biopsy	16S rNA sequencing	*Abiotrophia adiacens*	Ampicillin for 6 weeks	6 weeks	No	Cured
[8] (2010)	73, male	Back pain, fever	Hypertension, hyperlipidemia	No	Yes	L3-L4	Blood cultures	Vitek 2 system + 16S rNA sequencing	*Granulicatella adiacens*	Penicillin *G* + gentamicin for 6 weeks, po amoxicillin for 9 weeks	15 weeks	No	Cured
[14] (2005)	51, male	Back pain	Mitral valvulopathy, recent dental procedure	No	Yes	L2-L3, L5-S1, right sacroiliac joint	Blood cultures	16S rNA sequencing	*Abiotrophia defectiva*	Amoxicillin + gentamicin (stopped on day 5) + oral rifampicin, po amoxicillin for 11 weeks + po rifampicin for 10 weeks	14 weeks	No	Cured
[7] (2002)	68, male	Back pain, fever	Diabetes, coronary artery disease, infrarenal aorta prosthetic replacement, AF and AV block with VVI PM	Yes (PM lead)	Yes	T10-T11	Blood cultures	bioMérieux Rapid ID 32 Strep system	*Granulicatella adiacens*	Penicillin + gentamicin + rifampin	ND	No	Cured
[6] (1998)	45, male	Back pain, fever	ND	Yes	Yes	L2-L4	Blood cultures	ND	*Abiotrophia adiacens*	Penicillin for 2 weeks, po clindamycin for 2 weeks	4 weeks	No	Cured
[6] (1998)	50, male	Back pain, fever	ND	Yes	Yes	L3-L5	Blood cultures	ND	*Abiotrophia adiacens*	Penicillin + gentamicin for 2 weeks, ceftriaxone for 2 weeks	4 weeks	No	Cured

NA: not assessed; ND: not described; PM: pacemaker; AF: atrial fibrillation; AV: atrioventricular.

## Abbreviations

FDG-PET/CT: F18-fluorodeoxyglucose-positron emission tomography/computed tomography
IE: Infective endocarditis
iv: Intravenous
NVO: Native vertebral osteomyelitis
po: Per os
IQR: Interquartile range.
